# Pulmonary Fibrosis in Antineutrophil Cytoplasmic Antibodies (ANCA)-Associated Vasculitis: A Series of 49 Patients and Review of the Literature: Erratum

**DOI:** 10.1097/01.md.0000464203.25179.42

**Published:** 2015-01-16

In Figure 1 of the article “Pulmonary Fibrosis in Antineutrophil Cytoplasmic Antibodies (ANCA)-Associated Vasculitis: A Series of 49 Patients and Review of the Literature”,^1^ which appeared in Volume 93, Issue 24 of *Medicine*, the descriptions for panels B and C were originally reversed. The correct legend is given below. The article has since been corrected and reposted in the issue.

Representative high-resolution computed tomography showing typical features of the 3 major patterns of PF associated with AAV. Predominantly basal, subpleural reticular pattern with macrocystic honeycombing lesions in usual interstitial pneumonia (A). Centrilobular and paraseptal emphysema predominating in the upper lobes (B). Ground-glass opacities in a patchy distribution in nonspecific interstitial pneumonia (C), and reticular subpleural changes in the lower lobes, associated with few microcystic lesions suggesting honeycombing (D) in combined pulmonary fibrosis and emphysema syndrome.

## References

[1] Comarmond C Crestani B Tazi A Pulmonary fibrosis in antineutrophil cytoplasmic antibodies (ANCA)-associated vasculitis: A series of 49 patients and review of the literature. *Medicine* 2014; 93:340–349. 25500703 10.1097/MD.0000000000000217 PMC4602438

